# Opportunities and challenges in public and community engagement: the connected for cognitive health in later life (CHILL) project

**DOI:** 10.1186/s40900-018-0127-x

**Published:** 2018-11-19

**Authors:** Caroline Lee, Tom Mellor, Peggye Dilworth-Anderson, Tiffany Young, Carol Brayne, Louise Lafortune

**Affiliations:** 1 Cambridge Institute of Public Health, Forvie Site, University of Cambridge School of Clinical Medicine, Box 113, Cambridge Biomedical Campus, Cambridge, CB2 0SR UK; 2 Creative Research Collective, Cambridge, UK; 3 Gillings School of Global Public Health at the University of North Carolina Chapel Hill (UNC), Chapel Hill, USA; 4 North Carolina Translational and Clinical Science Institute, University of North Carolina Chapel Hill, Chapel Hill, USA; 5 Cambridge Institute of Public Health, Forvie Site, University of Cambridge School of Clinical Medicine, Cambridge, UK

**Keywords:** Public health, Community engagement, Public engagement, Participatory research, Risk factors, Dementia

## Abstract

**Plain English summary:**

Two goals of public health research are to understand what causes disease and ill health, and what can be done to prevent it. To develop appropriate and effective actions, we need to know what resources are available to communities, and what are the beliefs and values that influence behaviour. This means that research needs to be carried out close to the people it affects, to better understand context and environment, as well as people’s understandings and interpretations of health and health risk.

Connected for Cognitive Health in Later Life (CHILL) was a project developed to test whether engaging local residents in research might be a good way of firstly: raising awareness of research findings in the community; and secondly, affecting mid-life behaviours in favour of ageing well and reducing risk of dementia. We investigated perceptions of ageing and how to age ‘well’ in a town whose population health is ranked worse than the regional average. Project activities involved: identifying and engaging with stakeholders; conducting ‘mini’ street interviews; holding community workshops; and taking part in a large community event.

This paper describes the process of carrying out the research, and presents a flavour of some of the information captured on context and local understanding of dementia risk. It then goes on to discuss in more depth some of the challenges in attempting to involve people in shaping research and intervention development, before offering some conclusions and suggested next steps for researchers.

**Abstract:**

**Background**

Identifying risk of disease and ill health, and developing prevention strategies, are key objectives in public health research. However, poor understanding of the impact of local context, including cultural and ethnic differences, challenges our ability to develop actions that are acceptable and meaningful to local communities. This suggests a need for research embedded in sub-populations, seeking to better understand context, understanding and interpretation of health and health risk.

**Methods**

Against a backdrop of wide inequalities in health, the Connected for Cognitive Health In Later Life (CHILL) project began work in a locality with worse than regional average health outcomes aiming to co-develop a project investigating perceptions of ageing and how to age ‘well’. Another goal was to test the potential for using Community Based Participatory Research (CBPR) as a way of communicating research knowledge, raising awareness and understanding amongst community members of mid-life risk factors for developing dementia. A four-part scoping study was embarked on, including: stakeholder identification and engagement; street interviews; community workshops; and a wider public engagement event.

**Results**

Whilst the project was able to stimulate interest, gain involvement from a small group of residents, and successfully engage members of the public, it was not possible, within the relatively short timescale of the scoping project, to achieve the depth of community involvement necessary to co-design and seek additional funding for collaborative research activities.

**Conclusions**

A number of challenges were encountered in scoping CBPR on this particular topic and location. Potential explanations include lack of ‘readiness’ or ‘capacity’ amongst the local population, and a very limited timescale for the scoping research to adapt and respond to this. This has significant implications in terms of time and effort necessary to build infrastructure to support research partnerships if researchers wish to engage successfully with members of the public on population health in the future.

## Background

Identifying risk and developing prevention strategies are key objectives in public health research. Yet poor understanding of the impact of local context, or cultural and ethnic differences, challenges our ability to develop actions that are acceptable and meaningful to local communities. This suggests a need for research embedded in sub-populations, seeking to better understand context, understanding and interpretation of health and health risk.

Interest in place-based, and community engaged, approaches has been growing in national public health contexts, as evidenced by recent guidance issued by NICE and Public Health England (PHE) [1, 2]. Devolution in local government and budgetary challenges in all areas of public life further emphasise a policy shift and pragmatic service response focusing on prevention, self-management and local assets [3]. The research was inspired largely by this ‘place-based’ discourse, though we acknowledge that different definition and layers of ‘community’ exist, with individuals potentially belonging to several concurrently. Each layer, as well as intersectionality, are important to the creation of health in places.

Against a backdrop of wide inequalities in health, the Connected for Cognitive Health In Later Life (CHILL) study began work in a locality reporting worse than county-average health outcomes, such as poor self-reported health status and limitations in day-to-day activities [4], aiming to co-develop a project investigating perceptions of ageing and how to age ‘well’. Cambridgeshire, UK, has significant inequalities in health, with the district of Fenland having poorer outcomes than others [5]. In terms of issues in ageing, we now have sufficient evidence that *mid-life behaviours* influence risk of disability, dementia, and chronic non-communicable health conditions in later life [6]. Regional population statistics enable us to pinpoint the areas faring worse, for example in terms of health and disability in mid to late life, which in turn offers an opportunity to target messages about modifiable risk factors.

Following a period of consultation with regional and local stakeholders in health, public health, community engagement and third sector organisations, a locality within Fenland was selected to scope out feasibility of conducting contextualised and participatory research on this aspect of health. Whilst not the ‘worst’ performing in terms of health outcomes and deprivation indicators, the selected locality performed poorer than county average on many health indicators, and had not benefited from any investment in community engagement or place-based initiatives, unlike some ‘more deprived’ localities in the area. At the time of the scoping, an initiative grounded in building on community strengths or ‘assets’ was being launched in this and neighbouring towns, and we anticipated that the scoping study would dovetail with the introduction of Community Development Worker (CDW) support in engaging residents on the ground.

## Methods

The CHILL project aimed to assess the feasibility of adopting a Community Based Participatory Research (CBPR) approach to communicate knowledge from research around mid-life risk factors for dementia, and engage communities in making sense of that knowledge within their own lives, local context and choices around health and lifestyle. The intention was to explore and communicate research topics in ways that are more creative, inclusive and accessible than traditional research methods, e.g. employing diagramming, audio-visual material, and small focus groups. A longer term aim was to contribute to better description and understanding of the influence of community and beliefs on modifiable risk factors for dementia, and to co-develop responses (whether formal requiring resources, or informal ‘problem-solving’) reflecting local needs and assets.

The core output of the scoping phase (January to July 2016) was intended to establish a ‘working group’ of community members who would continue to work collaboratively to investigate these public health messages in the context of the everyday lives of residents. In the longer term, this group could potentially go on to co-develop local ‘interventions’ in support of cognitive health in later life, potentially accessing a newly available grant fund for community groups with the support of the CDW.

### Community based participatory research (CBPR)

CBPR is included in the collaboration and partnership branch of the PHE ‘family’ of community engaged approaches [2], yet there is limited experience of CBPR in UK public health research, with academic expertise concentrated primarily in the US [7, 8], developing countries, and often on a particular ethnic group [9, 10] or essentially ‘captive’ research communities, such as prisoners [11].

**Fig. 1:**
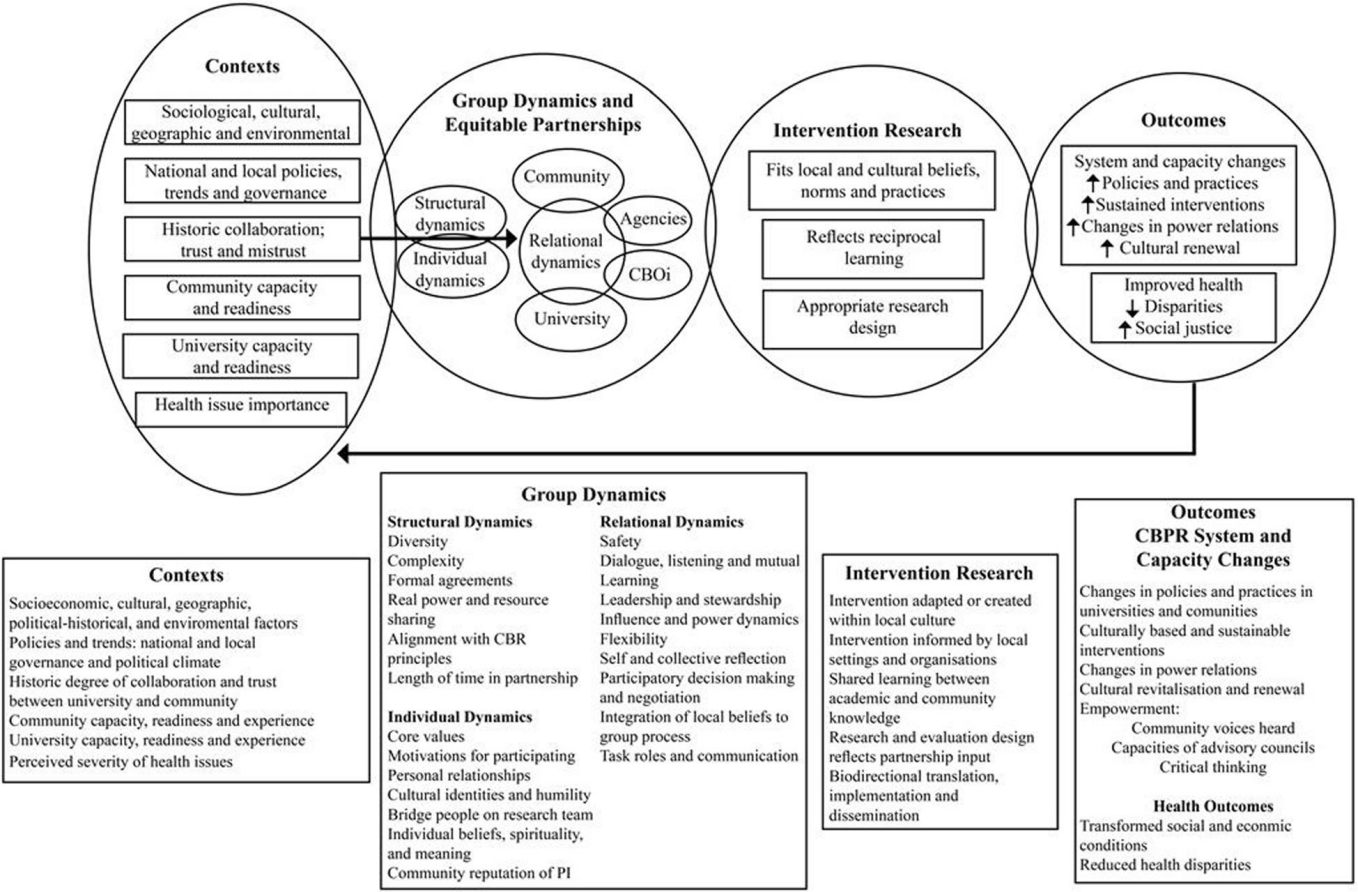
CBPR for health: a Logic Model

CBPR is described as *“The blending of principles and methods of scientific research with those of community organising”* ([8], pp136]). The approach encompasses mixed and varied methods which can be chosen to maximise engagement and co-production of the community in the research. Participatory Appraisal (PA) is another term associated with community-based research, and is closely aligned with CBPR. On the ground, PA activities typically foster communities generating their own knowledge and understanding through community assessment employing participatory methods [12]. For this reason, we refer to some of the exercises employed in the scoping project as aligned with a PA approach. PA implies that the expert-learner role is reversed between researcher and community member and advocates that the community is responsible for change [13]. CBPR, however, crucially involves partnership between communities, organisations *and* researchers, each sharing expertise and decision-making. As well as encompassing specific methodological principles, CBPR also represents a theory of change that anticipates the ‘community’ becoming mobilised to respond to a question through the research process itself. Thereby, both community and research process are agents and mechanisms of change. Figure 1 presents Wallerstein’s logic model for this [14], which was used to frame the scoping work.

Preliminary work on the ‘context’ aspects of this model began in the 9 months before on the ground activities took place, with a long period of intelligence gathering and consultation with regional and local public health, primary care, community health, ageing and dementia community and voluntary sector stakeholders. This fed into the background picture of policies and activities influencing public health and community engagement on the ground. Relevant initiatives had been pinpointed, with key developments in Asset Based Community Development (ABCD) [15, 16], in particular, a key influencer in selecting locality in anticipation of synergies, cooperation and information sharing on the ground. Reference to context continued throughout, and a group discussion was carried out with two key individuals employed by such initiatives to reflect on the experience of community development, engaging residents in behaviour change, and actions to engage with residents.

In attempting to engage residents in research findings and questions on a given topic, CHILL aimed to develop foundations for future participatory research with goals of transmitting evidence on dementia risk, and co-developing context-relevant responses. The scoping study was therefore structured according to three stages, all supportive of the first of the spheres (Context) illustrated in Fig. 1, and aimed to result in sufficient engagement to be moving to the second sphere (Group Dynamics and Equitable Partnerships), concerned with establishing partnerships.

#### Stage 1: Public perceptions and interest in topic

Despite the intention to undertake a participatory and empowering approach to research, there was an immediate paradox in that a research ‘topic’ of academic interest (risk factors for dementia in mid-life) had already been set for the project. This meant, rather than focusing on work with residents to identify their own priority topic, step one of ‘scoping’ instead involved stimulating public interest in the project, and in hearing research messages on the academic topic of interest. Researchers contacted and met stakeholders and frontline health promotion workers, local shops and businesses and key community organisations with possible reach to residents in mid-life (e.g. library and adult learning, community centres, secondary schools, Weightwatchers, Slimming World, and local churches). Access to important locations for publicising the project was negotiated through the Clinical Commissioning Group (CCG - statutory organisations of the English National Health Service responsible for planning and commissioning health care services for their local area), community pharmacy area lead, manager of the town supermarket, the leisure/fitness centre, and the town library. A visual presence was created, using a logo combining the project name with the town coat of arms. Leaflets and flyers were designed, a project Facebook and web page set up, − and publicity shared via two Facebook pages recommended by stakeholders as frequently accessed by residents, as well as the town’s newsletter.

#### Stage 2: Understanding context

Rapid street interviews, dubbed ‘Vox pops’, were conducted and audio recorded to gather a flavour of initial responses to key words and impressions of mid-life risk factors for dementia. Researchers asked a few short questions to capture people’s understanding of the terms ‘cognitive’, ‘brain health’ and ‘dementia’, as well as what they knew about modifiable risk and protective factors for dementia. Whilst ‘cognitive’ is not a term in common usage outside of clinical settings, we were interested in whether it was understood and prompted association with the brain, or brain training activities often promoted as protective of memory and recall. Rather than assuming it would not be understood, and limiting responses generated, both ‘brain’ and ‘cognitive’ were terms used as prompts during these exchanges.

Thirty five community based individuals and organisations were contacted about the project and its goal of engaging residents with research findings. In addition to project publicity, leafleting and social media, a long list of organisations and stakeholders were contacted and involved in discussions to assist access to local residents. These included: the leisure centre, community pharmacy branches, the GP surgery, the community centre, the library service, adult learning, early years education settings/nurseries, primary and secondary schools, the Fire and Police Services, Age UK, Alzheimer’s Society, The Rotary Club, the supermarket, the town council, The Working Men’s Club and The Conservative Club.

The second activity involved small group workshops with residents held in well-known and accessed community locations, both in the evening and weekend mornings. The purpose of the workshops was to hear about research evidence on risk factors for dementia, listen to local voices collected in the ‘Vox Pops’ in order to present a snapshot of local awareness and key questions, and introduce the CHILL project and initial purpose of engaging with local residents to think about research messages within a local context.

Creative approaches, in line with tools employed in PA to investigating local context ([2] pp25, [13]), were demonstrated as ‘taster’ exercises encouraging residents to think about different areas of risk highlighted by research in the context of the town where they lived. Asset mapping was also introduced, and participants engaged in diagramming some of the opportunities in the town in terms of key modifiable risk areas (e.g. diet, activity, social activity, lifelong learning and weight management). Town maps were distributed to help pinpoint key physical resources/assets, such as buildings and the location of key organisations and community groups. An adapted Strengths, Weaknesses, Opportunities and Threats (SWOT) analysis in relation to the Town’s capacity to support protective factors for healthy brain ageing was also carried out. The asset mapping and SWOT analysis provided some indicative data regarding social, cultural and environmental context for the community, as well as some insights into history of collaborative ventures, response to community consultation, and resulting trust or mistrust of attempts at engagement.

A second meeting using ‘body maps’ was held, exploring key influencers on individual behaviour and lifestyle choices (Fig. 5). The aim was to stimulate thinking about context and why research messages, even if increasingly well publicised in mainstream media, may not impact behaviour at individual and community levels.

Residents attending meetings became an informal working group of 12 for the CHILL project, communicating between face-to-face meetings via email.

#### Stage 3: Public engagement in research messages

**Fig. 2:**
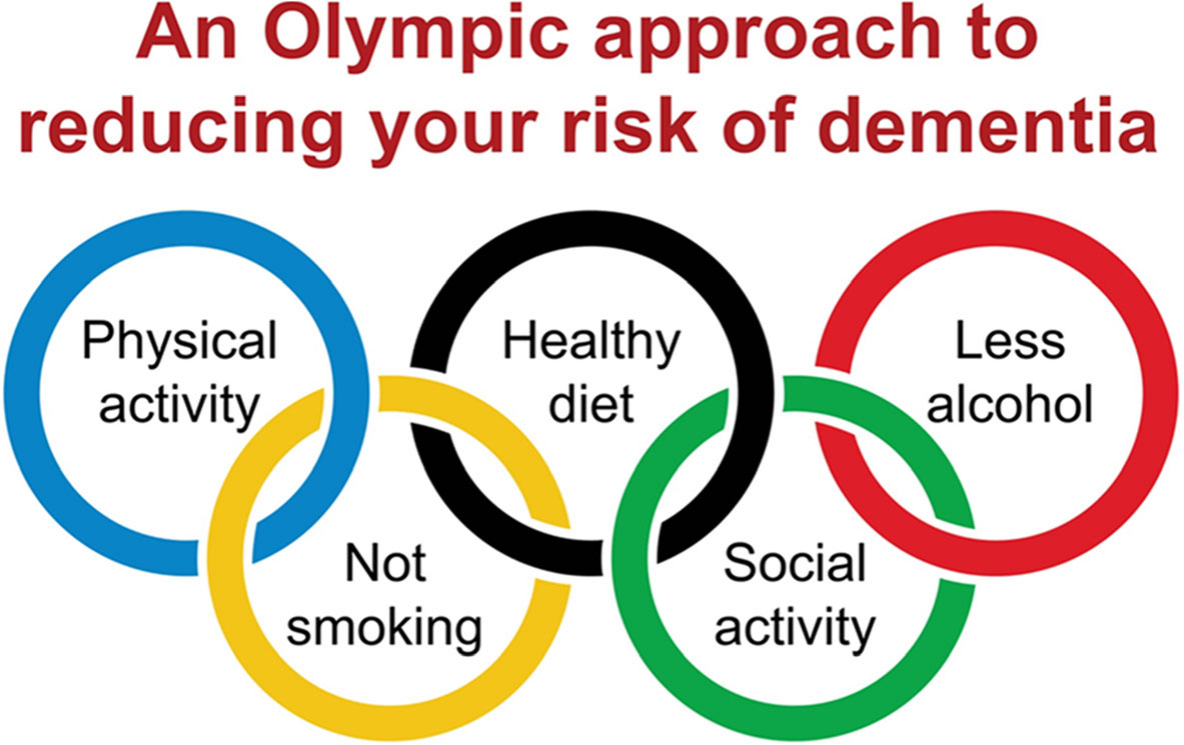
Mid-summer town festival poster/postcard

The final task of the scoping phase was to plan the project’s market stall at the mid-summer town festival. The group collaboratively agreed that the objective of the working group attending the festival was to share research evidence on risk with the wider public, to raise awareness of risk factors, and to garner interest in joining the CHILL working group. The group also determined that the mechanism for communicating the research messages should be fun and in keeping with a celebratory event. A smoothie bike (a stationary push bike fitted with a blender to make fruit drinks by pedal power) became the embodiment of two key behaviours to modify risk, healthy diet and physical activity, and the Rio Olympics theme of the festival was adopted into posters and postcards depicting the 5 Olympics rings (Fig. 2) as modifiable risk factors for dementia.

Through the establishment of a working group, the hope was for a fledgling ‘platform’ for CBPR to be developed, involving residents as co-researchers who could investigate commonly held beliefs around health, barriers and facilitators to lifestyle changes.

## Results

The following section presents our findings in relation to the key ‘scoping’ objectives of: baselining awareness and knowledge translation; understanding local context; and co-development in partnership with local people.

### Public engagement - awareness and knowledge translation

Two public engagement activities took place: one to gather a flavour of understanding and level of interest in the topic; and the second to communicate research messages on risk in a creative and engaging way. Both of these were successful in their objectives, as defined by depth of data gathered, and number of members of the public engaged with, numbers engaging in the core activity, and information distributed. The range of questioning was small, as detailed above, and data saturation was quickly reached from the Vox pops (*n* = 16), i.e. responses to the word prompts returned nothing new, and we were able to identify common questions and beliefs about cognitive health held amongst these local residents. *For example, in terms of awareness of cognitive health, dementia and mid-life risk factors, r*esearchers asked if people had questions about risk of dementia, and whether they wanted to know more. Many did:

*“What actually causes it? What causes brain cells to die away?”*

“*When my elderly father went into a care home, and you look around and you think well some of these people are really on the ball and others aren’t. What’s made the difference? What’s made some of them cope better and be able to stay more active and more ‘with it?”*

*“What is it that causes it, and why in some cases it obviously accelerates quicker than in others, and what we can do to prevent that?”*

Yet, there was also a minority who did not want to know more:

*“Not something I worry about – if things are ok I don’t tend to worry about it.”*

### Community workshops - understanding context

**Fig. 3:**
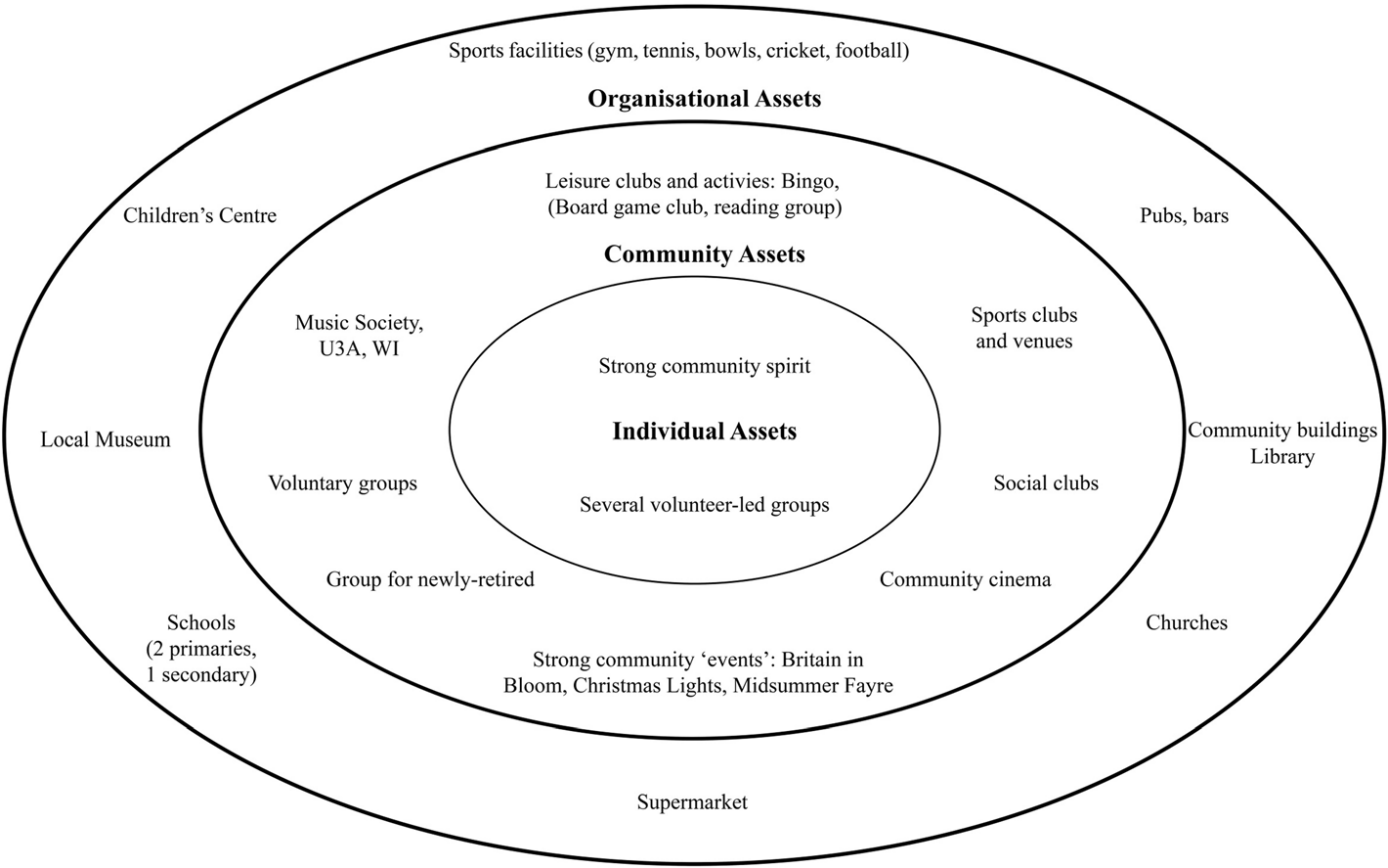
Asset mapping exercise

**Fig. 4:**
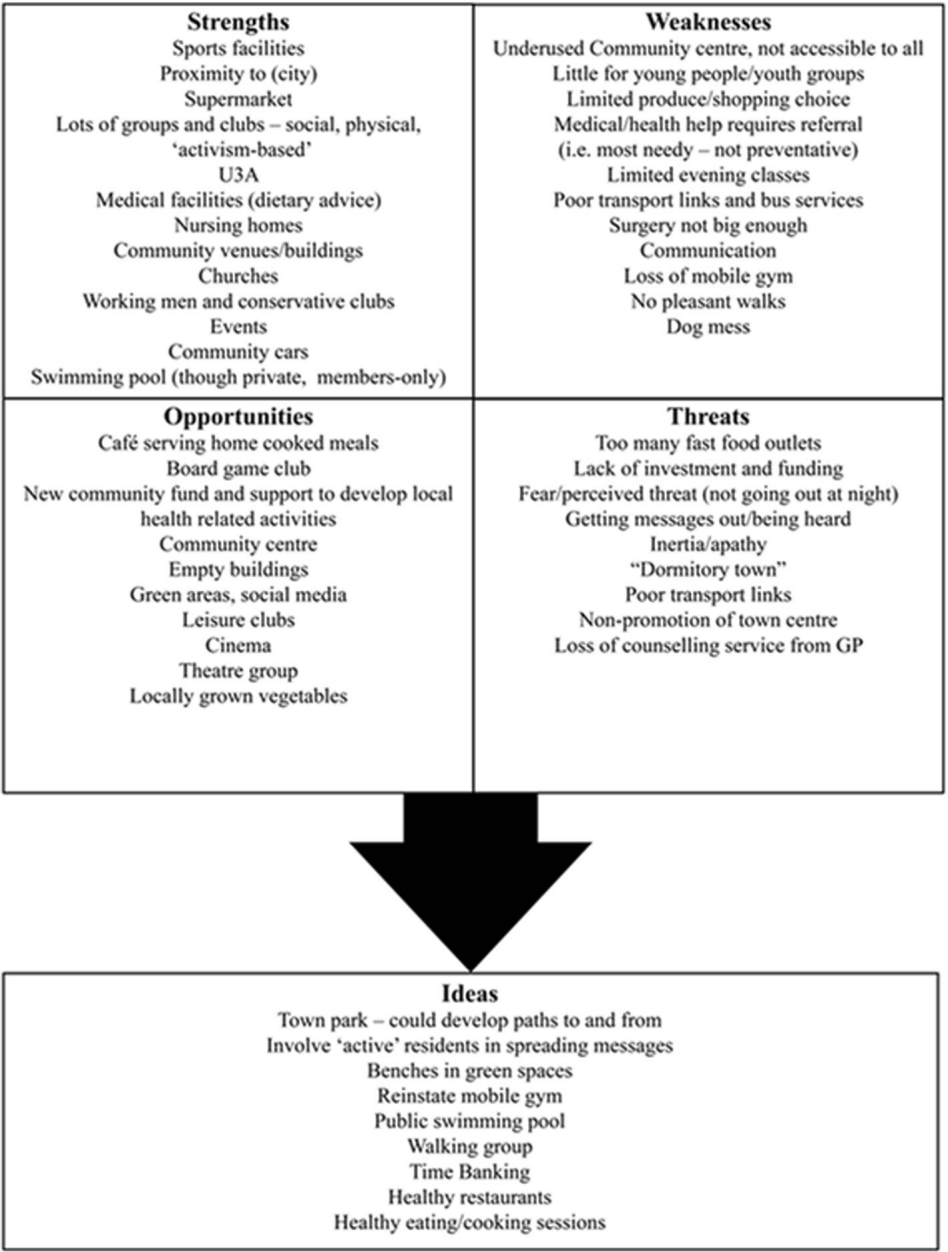
‘SWOT’ analysis (local assets and healthy ageing)

**Fig. 5:**
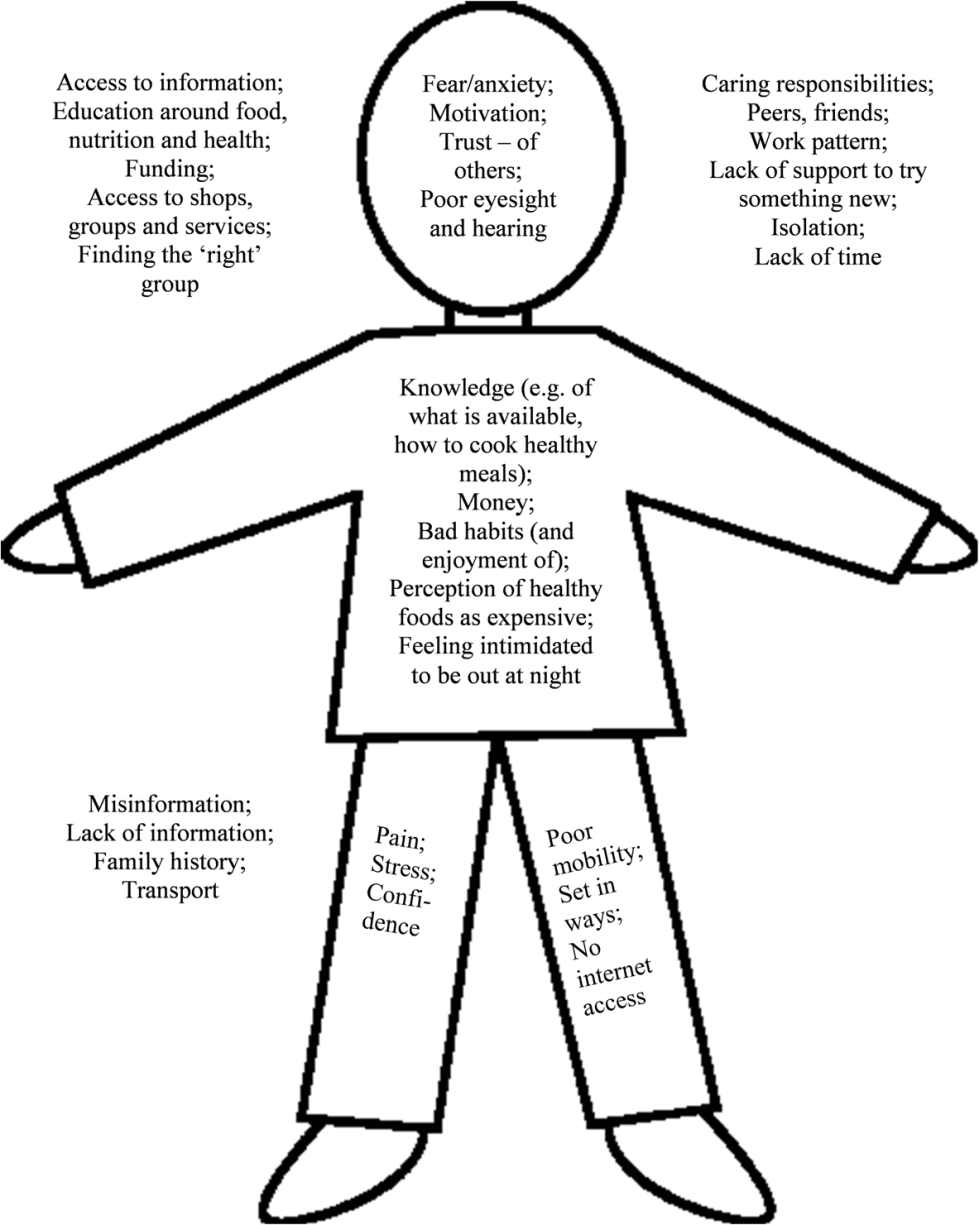
Body mapping exercise

The community workshops that followed the Vox Pop exercise were attended by two small groups of local residents (workshop 1, *N* = 11, workshop 2, *N* = 5) and the exercises carried out provided tasters of activities typical of a Participatory Appraisal (PA) process, as well as producing indicative data on local context and culture. Figures 3 and 4 illustrate ideas generated by the asset-mapping and SWOT exercises carried out by residents considering the local resources that could be protective of brain health, challenges in the town, what was supportive to health, and where there may be opportunities to do more. Figure 5 illustrates the second-stage exercise aiming to encourage residents to think about the interaction between ‘internal’ and ‘external’ influencers on health promoting behaviours, including e.g. place/context, beliefs, and family and relationships.

Whilst the above provide some insight into local context and influencers, the numbers reached were small and hence, results cannot be assumed to be representative of the local mid-life community as a whole.

### Engagement and co-development

In addition to presenting research evidence on risk and to exploring community responses, the community workshops had the additional purpose of stimulating interest in engaging in research as a working group. Even though efforts were made to publicise the project and community workshops through locally-supported venues and channels of communication, the majority of residents who attended the workshops were reached via face to face meetings, and via the Vox Pop exercise. Strong links resulted from meeting with the town council and key individuals with a community engagement and health promotion remit on the ground.

At the outset, we had hoped that the scoping study would open the door to community members becoming peer-researchers to survey residents and gather data aiming to describe the influence of community and culture on modifiable risk factors for dementia – i.e. to address context. However, without some pre-existing foundations of engagement to build on, our expectations were unrealistic within timescale and resources. As noted in the Communities in Control study, an evaluation of a UK place-based initiative of resident needs-led action called Big Local, the extent and speed of collective action in support of public health is heavily influenced by a number of factors. These include;: the relative ‘starting points’; the cohesiveness of local identities’; and the presence of community activists and existing organisations and structures [17, 18]. This suggests, therefore, that some localities require more time to build connections and structures in support of articulating their own priorities than others. Even though CDWs had been appointed to work in the area during the scoping phase, they did not start until mid-way through the project, and as they were not local residents, were also starting engagement activities from scratch.

Our limited success in engaging residents as a formalised working group (the foundations to CBPR), perhaps reflects that some of these building blocks were not well developed in this locality. Key voices in the community themselves reflected that it was frequently the ‘same people’ involved in everything. This suggests that, despite the town apparently having a range of activities to offer (as indicated by workshop ‘asset mapping’ exercises), the existing groups and associations rely heavily on a core of individuals, and do not necessarily reach members of the wider community.

Evidence for this potential lack of ‘readiness’ or ‘capacity’ for engagement was highlighted in a group discussion between the lead researcher and two community engagement workers as one of a number of challenges encountered in engaging this community.

*“Challenges of raising awareness, getting word out that there’s a new project, then getting commitment and willingness to take responsibility – people don’t want that, they don’t want to be part of a committee” (Community Development Worker 1)*

*“Hard to promote what we’re doing – it’s a free service but it is hard to get engagement – healthy eating groups – no one wants to book in” (Community Development Worker 2)*

An obvious additional tension, not to be under-emphasised, lies between the premise in CBPR that research direction and priorities should be agreed with the community, the relatively limited engagement with residents had been achieved, and the practice that this ‘research idea’ ultimately belonged to the academic ‘partner’. To what extent was the research topic one deemed to be a priority by residents, if at all? What research priorities did they have? Paradoxically, in adhering as closely as possible to an ethos of co-production and co-design in shaping the direction of CHILL, and encouraging the group to set their own agenda, we discovered that asking people to engage with researchers in something not yet well defined was extremely challenging. Co-construction of what to study and focus research on was an unfamiliar way of working, with residents seemingly more familiar or comfortable with direction than collaboration.

Pursuing the goal of jointly agreed priorities with those who were ‘at the table’ with CHILL was also difficult due to limited capacity *–* the individuals engaging with the project already having several professional and voluntary commitments, and little to no spare time or energy for new ventures.

Key informants working in Community Development confirmed that it was difficult to engage residents in the locality both in services and in health-related projects, and especially so for a 40–65 year old ‘sandwich’ generation, who not only tend to be working, but also have caring responsibilities both for children and older parents.

*“You get more engagement from the older generation – mid-life is difficult to engage with.” (CE worker 2)*

A member of the research team also reflected on this reticence:

*“The easy bits are finding out what people’s questions are (about dementia), bringing people together and giving some research messages. But the concept of then steering a group, deciding where to go with it, you can see people pulling back from that idea physically.” (Researcher/facilitator)*

The ideas generated in the community workshops from the asset mapping and SWOT analysis exercises enabled residents to highlight community assets and begin to reflect on gaps and opportunities to develop local resources and activities in support of brain health/reduction of mid-life risk factors. This indicated that there was scope for, and potential interest among, local residents to engage in PA methods, whether or not as part of a broader programme of CBPR. However, deeper engagement with sufficient numbers of residents to drive a working group was a difficult next step to bridge. This meant that ultimately, it was not possible in this locality at this time to develop beyond the scoping project, into a further piece of work embedding a CBPR partnership to address risk factors for dementia in mid-life.

## Discussion

Three inter-related challenges can be identified in the CHILL experience: 1/ limited engagement of residents (numbers reached and range of interest groups represented); 2/ lack of ‘readiness’ or ‘capacity’ to drive research or action; and, 3/ lack of infrastructure in support of collaborative research or action with residents.

All of these are central tenets to successful community engagement, as highlighted by the research and methodology literature [19]. For example, the Scottish Community Development Centre reported on the importance of community infrastructure and existing groups to successful community engagement ([20] pp3, [21]):

*“The quality of the community engagement process is affected by the level of existing community infrastructure and the availability of groups to engage with. Where there is an element of community infrastructure in place, quality of process is, in turn, affected by the community groups’ ability to engage with the wider community and a diverse range of interests.”*

The Communities in Control study highlights three indicators of ‘collective control’ which are pertinent to our experience in scoping CBPR [22]. 1/ The ‘Power to’, which can be seen as a virtual or physical ‘infrastructure’- the existence or *“creation of organizational structures and arrangements that enable people to come together in collective decision making and direct action”*; 2/ the ‘Power within’, which maps to ideas of capacity, the “*development of skills, confidence and critical awareness at a group level that equip people with the ability and drive to take action together*”; and 3/ the ‘Power with’, mapping to establishing and growing “*links and alliances that develop with other groups or organisations in pursuit of common goals or interests”* ([22], p1]).

Public Health England brought out national guidance on community centred approaches in 2015 [2], offering a ‘family’ of practical evidence-based approaches with potential to produce evidence and practical information in support of commissioning and practice. The experience of CHILL suggests that some elements in the family are perhaps only feasible once other elements have occurred. Specifically, in the case of CBPR, we observe a need to attend to community infrastructure before collaboration and partnerships *for co-productive research* can occur. Essentially, this refers both to the concept of community ‘readiness’, and ‘capacity’, described in the literature as critical for effective engagement activity to progress. This assertion is supported by Letcher and Perlow in their CBPR study embedded within a pre-existing Time Bank [23], and by the evaluation of the Communities that Care programme [24].

A criticism of the CHILL project can certainly be levelled at the pre-definition of research focus, and this makes it challenging to assess the influence of this immediate constraint on engagement with residents in the locality against the assertions made around ‘readiness’ and infrastructure. Nevertheless, we would argue there is sufficient literature around challenges 2 and 3, highlighted above and below, to support our evidence suggesting that elements of these were at play.

Much of the CBPR literature [21] is grounded either in the US experience or developing countries, and it may be that funding programmes and relative advancement of asset based approaches to community development, compared to the UK, has created a foundation for this kind of work in certain communities of interest. It may also be that place-based community remains stronger in some parts of the world compared to other areas. In some areas ‘community’, in the sense of ‘neighbourliness’ and willingness to ‘pull together’, is often said to have been eroded, whilst in other parts, local residents are mobilising their assets, and volunteering is strong. Despite some strong grass roots action, in England, communities (however defined) outside large and well-resourced initiatives such as ‘Big Local’ or ‘Well London’ [25, 26] may nevertheless be unused to being asked to direct or co-produce new activities, much less to come up with research questions and priorities for investigation. Hence, there may be a considerable gap between what a community engaged research approach expects from the public, and what people are prepared to take on.

So, where does this leave us in terms of identifying risk and developing prevention strategies? How do we ensure in the future we can move beyond the relatively attainable objectives of communicating research, raising awareness and understanding of dementia risk, towards a better understanding of locality-sensitive influences on behaviour, and the development of appropriate place-based interventions?

Is a minimum level of ‘readiness’ or ‘capacity’, in all partners, an essential starting point for undertaking CBPR? The Community Development world has moved on to embrace ABCD, the clinical world to embrace Patient and Public Involvement (PPI) [27], and public health research to see the value, legitimacy and potential impact of community-engaged research. Yet, it cannot be assumed that residential areas and ‘communities’ have been on the same journey, and there is potentially a mismatch. The challenge now, if we believe in the ability of CBPR to deliver on the longer term goals, is to find the right bridge for the gap observed here, and likely in many other UK and international localities.

It seems sensible to suggest that an assessment of the level of exposure and involvement of the population or community of interest to community based research/PA activities early on in the project is needed to determine the level of work needed to build partnerships. We could further argue that there needs to be discussion about integrating an additional ‘middle stage’ in community engaged development and research initiatives which reflects a recognition that embedding meaningful patient and public involvement (PPI) in public health research has taken considerable time, training and resources. This middle stage in community-centred, participatory public health research may require activities and resources to support residents, communities and researchers together to agree the role of community members in public health, especially if it is to be actively engaged in decision-making, developing and directing activities, rather than as passive receivers of increasingly limited statutory services.

This rationale also brings us to a final limitation of the scoping study - timeframe. While methodological texts tend to describe CBPR as a drawn-out process and long term commitment [8], CHILL had relatively limited research capacity and timeframe from concept to end of scoping – approximately 18 months - a challenging timeframe given the range of limitations described above.

At the end of this scoping work, we are left with a question. If we recognise the importance of infrastructure and capacity for effective CBPR on broad public health research questions, or community engaged research within localities, will research funding follow that secures researchers time and resources (perhaps alongside community development workers) to identify local infrastructure, listen to local people, raise awareness of the value of research, and create capacity to conduct or engage with it?

## Conclusions

The investment in this scoping study was worthwhile in contributing to a better understanding of opportunities and challenges of aspiring to community engaged and participatory work with new ‘research communities’. The alignment of work to the preliminary stages in a CBPR logic model was helpful in generating a depth of understanding and concern over dementia risk, reaching residents with research messages around mid-life risk factors for dementia, and carrying out elements of Participatory Appraisal, such as mapping local assets according to what is supportive of, or a challenge to, cognitive health. Community engagement in hearing and interpreting research findings, although ‘light touch’ by involvement definitions, enables relationships between researchers and residents to develop, identifying those interested and in a suitable position to engage. Developing the level of engagement further into co-design, community research and ultimately co-development of local initiatives in support of cognitive health was beyond the capacities both of the project and of the community at this time. Although integral to CBPR, without infrastructure or without additional capacity for engagement within existing arrangements, we find ourselves stymied at the ‘foundation’ stage. Hence, our conclusions are threefold:

- We have a better understanding and recognition of the importance not just of ‘foundations’ for CBPR, but also the work and time required to build capacity and trust in some UK communities to want to engage more deeply with researchers. (In this case on dementia risk, but also potentially on prevention of other health issues and conditions); and
- (in this instance) there was a lack of infrastructure and readiness/capacity for engagement in research.
- In order to move forward with community engaged or participatory research at a ‘whole community’ (as opposed to interest or patient-group) level, there seems to be a need for a physical or virtual ‘infrastructure’ for community engagement in research that all sectors of the community could become involved with.

These findings are important for both research and community development practitioners aiming to engage communities with little history of participation (other than perhaps as service users) with statutory or voluntary sectors and participatory approaches in broad action, such as on inequalities or whole-population behaviours.

## Abbreviations

ABCD: Asset Based Community Development
CBPR: Community Based Participatory Research
CCG: Clinical Commissioning Group
CDW: Community Development Worker
CHILL: Connected for Cognitive Health in Later Life
CIC: Communities In Control
PA: Participatory Appraisal
SWOT: Strengths Weaknesses Opportunities Threats

## References

[1] National Institute for Health and Care Excellence (2016). Community Engagement: Improving Health and Wellbeing and Reducing Health Inequalities [NG44].

[2] South J (2015). A guide to community-centred approaches for health and wellbeing.

[3] NHS (2014). Five year forward View.

[4] https://fingertips.phe.org.uk/profile/health-profiles/data#page/1/gid/1938132696/pat/6/par/E12000006/ati/101/are/E07000010. (Accessed 25 Feb 2016).

[5] Cambridgeshire Annual Public Health Report 2017. Cambridgeshire County Council. https://cambridgeshireinsight.org.uk/wp-content/uploads/2017/08/Cambridgeshire-Annual-Public-Health-Report-2017.pdf. (Accessed 25 Sept 2018).

[6] Lafortune L, Martin S, Kelly S (2016). Behavioural risk factors in mid-life associated with successful ageing, disability, dementia and frailty in later life: a rapid systematic review. PLoS One.

[7] Ramsden VR, Mckay S, Bighead S (2014). Engaging with the community to enhance primary health care. In promoting change through action research (207–34).

[8] Isreal B, Eng E, Schulz A, Parker E (2005). Methods in community-based participatory research for health.

[9] Karmaliani R, McFarlane J, Asad N (2009). Applying community-based participatory research methods to improve maternal and child health in Karachi, Pakistan. Nurs Outlook.

[10] Ramsden VR, McKay S, Patrick K, Bourassa C, Crowe J, Sanderson PK (2010). Community based participatory project: engaging individuals/families in the development of programs to enhance health and well-being of the Métis nation— Saskatchewan. AHTF—final report.

[11] Martin RE, Murphy K, Chan R (2009). Primary health care: applying the principles within a community-based participatory health research project that began in a Canadian women’s prison. Glob Health Promot.

[12] Chambers R. Relaxed and Participatory Appraisal (1996). Notes of practical approaches and methods.

[13] Williams KJ, Gail Bray P, Shapiro-Mendoza CK, Reisz I, Peranteau J (2009). Modeling the principles of community-based participatory research in a community health assessment conducted by a health foundation. Health Promot Pract.

[14] Wallerstein N, Oetzel J, Duran B, Tafoya G, Belone L, Rae R (2008). What predicts outcomes in CBPR? In community based participatory research for health: process to outcomes (371–92).

[15] Foot J, Hopkins T. A glass half-full: how an asset approach can improve community health and well-being. Great Britain Improvement and Development Agency 2010.https://www.local.gov.uk/asset-approach-community-wellbeing-glass-half-full. (Accessed 5 Mar 2017).

[16] Russell C. Asset-Based Community Development (ABCD): Looking Back to Look Forward: In conversation with John McKnight about the intellectual and practical heritage of ABCD and its place in the world today. (eBook) 2015. https://itunes.apple.com/GB/book/id1007493751. (Accessed 10 Sept 2017).

[17] CiC Study Project Briefing #3. How social context influences resident led action in an area based empowerment initiative. https://sphr.nihr.ac.uk/wp-content/uploads/2018/08/CiC-Bites3-Social-Context.pdf. (Accessed 7 May 2017).

[18] NIHR School for Public Health Research. Communities in Control Study – What are we learning? http://localtrust.org.uk/assets/downloads/documents/Communities%20in%20control%20-%20What%20are%20we%20learning%20final.pdf. (Accessed 7 May 2017).

[19] Harden A, Sheridan K, McKeown A, Dan-Ogosi I, Bagnall AM (2015). Evidence review of barriers to, and facilitators of, community engagement approaches and practices in the UK.

[20] Case Studies in Community Engagement within the Context of Community Safety (2013). Project analysis report Scottish Community Development Centre.

[21] Jagosh J, Bush PL, Salsberg J (2015). A realist evaluation of community-based participatory research: partnership synergy, trust building and related ripple effects. BMC Public Health.

[22] CiC Study Project Briefing #2: Identifying indicators of ‘collective control’ in an area based empowerment initiative. https://sphr.nihr.ac.uk/wp-content/uploads/2018/08/CiC-Bites2-Collective-Control-1.pdf. (Accessed 7 May 2017).

[23] Letcher AS, Perlow KM (2009). Community-based participatory research shows how a community initiative creates networks to improve well-being. Am J Prev Med.

[24] Crow I, France A, Hacking S, Hart M. The evaluation of the ‘communities that care’ demonstration projects. York: The Joseph Rowntree Foundation; 2004. https://www.jrf.org.uk/report/evaluation-three-communities-care-demonstration-projects. Accessed 25 Feb 2017.

[25] http://localtrust.org.uk/. (Accessed 25 Feb 2017).

[26] http://www.welllondon.org.uk. (Accessed 7 Nov 2017).

[27] http://www.nihr.ac.uk/patients-and-public. (Accessed 5 Dec 2017).

